# Impact of socioeconomic status on survival of colorectal cancer patients

**DOI:** 10.18632/oncotarget.20859

**Published:** 2017-09-13

**Authors:** Qian Zhang, Yufu Wang, Hanqing Hu, Rui Huang, Lei Xie, Enrui Liu, Ying-Gang Chen, Guiyu Wang, Xishan Wang

**Affiliations:** ^1^ Department of Colorectal Surgery, The Second Affiliated Hospital of Harbin Medical University, Harbin, Heilongjiang 150086, PR China; ^2^ Colorectal Cancer Institute of Harbin Medical University, Harbin, Heilongjiang 150086, PR China; ^3^ Department of Colorectal Surgery, Cancer Hospital of Chinese Academy Sciences, Beijing, 100021, PR China

**Keywords:** socioeconomic, SEER, colorectal cancer, survival, adverse

## Abstract

Socioeconomic status (SES) has an impact on the survival of various cancers, but it has not been fully understood in colorectal cancer (CRC). The Surveillance, Epidemiology and End Results database was adopted to detect the role of SES in the survival outcomes of CRC. A total of 184,322 eligible patients were included and SES status was analyzed. The multivariable analysis showed that Non-Hispanic Black (HR, 1.20; 95% CI, 1.15–1.24), being widowed (HR, 1.04; 95% CI, 1.01–1.07), any Medicaid (HR, 1.36; 95% CI, 1.33–1.39) and the lowest education level group patients had relative poorer prognosis. Besides, sex, tumor location, age, differentiation level and American Joint Committee on Cancer stage also had significant effects on overall survival of CRC. The individuals were further divided into five groups according to the number of survival-adverse factors. All of the four groups containing adverse factors showed impaired survival outcomes compared with the group containing no adverse factor.

## INTRODUCTION

Colorectal cancer (CRC) is the third most common malignancy in the United States, with 135,430 new cases and 50,260 deaths expected in 2017 [1]. The survival of patients with CRC have improved substantially due to the development of operation skills, chemotherapy drugs, immune treatment and early screening methods including timely colonoscopy examination, blood and fecal test and CT scan, etc. However, the survival rate of patients with CRC has changed little over the past 10 years.

The outcome of CRC is determined by various factors, including American Joint Committee on Cancer AJCC staging, tumor location, and neoadjuvant/adjuvant chemotherapy. Besides, socioeconomic status (SES) is also an important prognosis factor of CRC. Previous data indicated that married individuals possess better prognosis for many major causes of death compared with those who are single, separated, widowed, or divorced [2–4]. In consider of high complexity, length of duration, high cost of cancer-related therapies and the complicated role of SES in CRC survival, we hypothesized that SES factors, including insurance status, marital status, income, and educational level, may contribute to the overall survival (OS) observed in patients with colorectal cancer.

In this study, we used data from the Surveillance, Epidemiology and End Results (SEER) diagnosed between 2007 and 2013 to investigate the impact of SES on OS of CRC in detail.

## RESULTS

### Patient baseline characteristics

A total of 184,322 eligible patients were included, including 98,802 (53.6%) male and 85,520 (46.4%) female patients. The median age was 67 years old with the range 20–108 years old. The median follow-up time was 24 months. Among them, 15436 (8.4%) had well differentiation, 113682 (61.7%) moderate differentiation, 26670 (14.5%) poor differentiation, 3661 (2.0%) un-differentiation and 24839 (13.5%) unknown differentiation. The tumor site distribution was as follows: 128342 (69.6%) colon (with 38912 right colon and 14762 left colon), 48676 (26.4%) rectum and 6157 (3.3%) large intestine, NOS (not specified). Among these individuals, 43788 (23.8%) were stage I, 44223 (24.0%) stage II, 45857 (24.9%) stage III and 34637 (18.8%) were stage IV. We also analyzed the race distribution: 19108 (10.4%) were Hispanic, 126482 (68.6%) Non-Hispanic White, 21201 (11.5%) Non-Hispanic Black, 1121 (0.6%) Non-Hispanic American/Alaska native, and 15767 (8.6%) were Non-Hispanic Asian or Pacific Islander. Most of the included patients were married (104562, 56.7%), and single, widowed, and divorced patients were 29172 (15.8%), 32370 (17.6%) and 18218 (9.9%) respectively. The cohort included 155881 (84.6%) insured patients, 22111 (12.0%) any-Medicaid patients and 6330 (3.4%) uninsured patients. Moreover, the characteristics were stratified by sex. It showed that there was a dramatic difference between male and female individuals. Other data including SEER registry, histology, income and education level were also listed in Table 1.

**Table 1:** Characteristics of patients with CRC at the time of diagnosis in the US SEER, 2007 to 2012

Characteristics	Total No. (%)	Male	Female	*P* value
**Sex**	184,322 (100.0)	98802 (53.6)	85520 (46.4%)	
**Age [Median(range)], y**	67 (20–108)	60 (20–107)	69 (20–108)	< 0.001
Quartile 1 (< 56)	42482 (23.0)	23196 (12.6)	19286 (12.4)	
Quartile 2 (56–66)	46869 (25.4)	27601 (15.0)	19268 (10.4)	
Quartile 3 (67–77)	48094 (26.1)	26799 (14.5)	21295 (11.6)	
Quartile 4 (> 77 )	46877 (25.4)	21206 (11.5)	25671 (13.9)	
**Follow-up time [Median(range)], Mo**	24 (0–83)	24 (0–83)	24 (0–83)	< 0.001
Quartile 1 (< 8)	43716 (23.7)	23232 (12.6)	20484 (11.1)	
Quartile 2 (8–23)	46554 (25.3)	25354 (13.8)	21200 (11.5)	
Quartile 3 (24–47)	47009 (25.5)	25220 (13.7)	21789 (11.8)	
Quartile 4 (> 47)	47043 (25.5)	24996 (13.6)	22047 (12.0)	
**Differentiation no. (%)**				< 0.001
Well	15436 (8.4)	8265 (4.5)	7171 (3.9)	
Moderate	113682 (61.7)	61733 (33.5)	51949 (28.2)	
Poor	26670 (14.5)	13340 (7.2)	13330 (7.2)	
Undifferentiation	3661 (2.0)	1739 (0.9)	1922 (1.0)	
Pre-b	916 (0.5)	561 (0.3)	355 (0.2)	
Unknown	23923 (13.0)	13141 (7.1)	10782 (5.9)	
**Site no. (%)**				
Colon	128342 (69.6)			
Right colon	38912 (21.1)	18345 (9.9)	20567 (11.2)	0.000
Left colon	14762 (8.0)	8021 (5.4)	6741 (2.6)	
Others	74668 (40.5)			
Rectum	48676 (26.4)	28433 (15.4)	20243 (11.0)	
Large intestine, NOS	6157 (3.3)	3153 (1.7)	3004 (1.6)	
**AJCC stage, no. (%)**				0.000
I	43788 (23.8)	23629 (12.8)	20159 (10.9)	
II	44223 (24.0)	23305 (12.6)	20918 (11.3)	
III	45857 (24.9)	24306 (13.2)	21551 (11.7)	
IV	34637 (18.8)	19343 (10.5)	15294 (8.3)	
Unstaged	15817 (8.6)	8219 (4.5)	7598 (4.1)	
**Race no. (%)**				0.000
Hispanic	19108 (10.4)	10696 (5.8)	8412 (4.6)	
Non-Hispanic White	126482 (68.6)	67820 (36.8)	58662 (31.8)	
Non-Hispanic Black	21201 (11.5)	10861 (5.9)	10340 (5.6)	
Non-Hispanic American/Alaska native	1121 (0.6)	602 (0.3)	519 (0.3)	
Non-Hispanic Asian or Pacific Islander	15767 (8.6)	8460 (4.6)	7307 (4.0)	
Other	643 (0.3)	363 (0.2)	280 (0.2)	
**Histology no. (%)**				0.000
Mucinous adenocarcinoma	12010 (6.5%)	6084 (3.3)	5926 (3.2)	
Signet ring cell carcinoma	1710 (0.9%)	923 (0.5)	787 (0.4)	
Carcinoid tumor	6456 (3.5%)	3211 (1.7)	3245 (1.8)	
Other	164146 (89.1%)	88584 (48.1)	75562 (41.0)	
**Registry no. (%)**				0.000
San Francisco-Oakland SMSA	9739 (5.3)	5057 (2.7)	4682 (2.5)	
Connecticut	8418 (4.6)	4400 (2.4)	4018 (2.2)	
Detroit (Metropolitan)	9386 (5.1)	4819 (2.6)	4567 (2.5)	
Hawaii	3598 (2.0)	2082 (1.1)	1516 (0.8)	
Lowa	8617 (4.7)	4503 (2.4)	4114 (2.2)	
New Mexico	3768 (2.0)	2115 (1.1)	1653 (0.9)	
Atlanta (Metropolitan)	5582 (3.0)	3005 (1.6)	2577 (1.4)	
San Jose-Monterey	4544 (2.5)	2446 (1.3)	2098 (1.1)	
Los Angeles	19336 (10.5)	10349 (5.6)	8987 (4.9)	
California excluding SF/SJM/LA	40031 (21.7)	21667 (11.8)	18364 (10.0)	
Kentucky	12592 (6.8)	6852 (3.7)	5740 (3.1)	
Louisiana	11946 (6.5)	6587 (3.6)	5359 (2.9)	
New Jersey	20202 (11.0)	10437 (5.7)	9765 (5.3)	
Greater Georgia	13657 (7.4)	7451 (4.0)	6206 (3.4)	
Utah	3412 (1.9)	1894 (1.0)	1518 (0.8)	
Seattle (Puget Sound)	8849 (4.8)	4780 (2.6)	4069 (2.2)	
Rural Georgia	367 (0.2)	217 (0.1)	150 (0.1)	
**Marital Status no. (%)**				0.000
single (never married)	29172 (15.8)	16389 (8.9)	12783 (6.9)	
Married (including common law)	104562 (56.7)	65531 (35.6)	39031 (21.2)	
Widowed	32370 (17.6)	8026 (4.4)	24344 (13.2)	
Divorced	18218 (9.9)	8856 (4.8)	9362 (5.1)	
**Insurance status no. (%)**				0.000
Insured	155881 (84.6)	84222 (45.7)	71659 (38.9)	
Any Medicaid	22111 (12.0)	10890 (5.9)	11221 (6.1)	
Uninsured	6330 (3.4)	3690 (2.0)	2640 (1.4)	
**County-level income at time of diagnosis no. (%)**^a^				0.000
Quartile 1 (< US $48,540)	46193 (25.1)	25152 (13.6)	21041 (11.4)	
Quartile 2 (US $48,550–55,870)	47580 (25.8)	25568 (13.9)	22012 (11.9)	
Quartile 3 (US $55,870–68,520)	44519 (24.2)	23839 (12.9)	20680 (11.2)	
Quartile 4 (> US $68,520)	46030 (25.0)	24243 (13.2)	21787 (11.8)	
**County-level education no. (%)**^b^				0.000
Quartile 1 (< 21.06%)	45174 (25.1)	25536 (13.9)	20638 (11.2)	
Quartile 2 (21.07–29.91%)	55138 (29.9)	29494 (16.0)	25644 (13.9)	
Quartile 3 (29.92–35.57%)	37113 (20.1)	19722 (10.7)	17391 (9.4)	
Quartile 4 (> 35.57%)	45897 (24.9)	24050 (13.0)	21847 (11.9)	

Abbreviations: CRC indicates colorectal cancer; SEER, Surveillance, Epidemiology, and End Results.

^a^Counties ranked by median household income in US $10.

^b^Counties ranked by the percentage of adult individuals with a Bachelor's degree.

### Impact of socioeconomic status on survival

**Figure 1:**
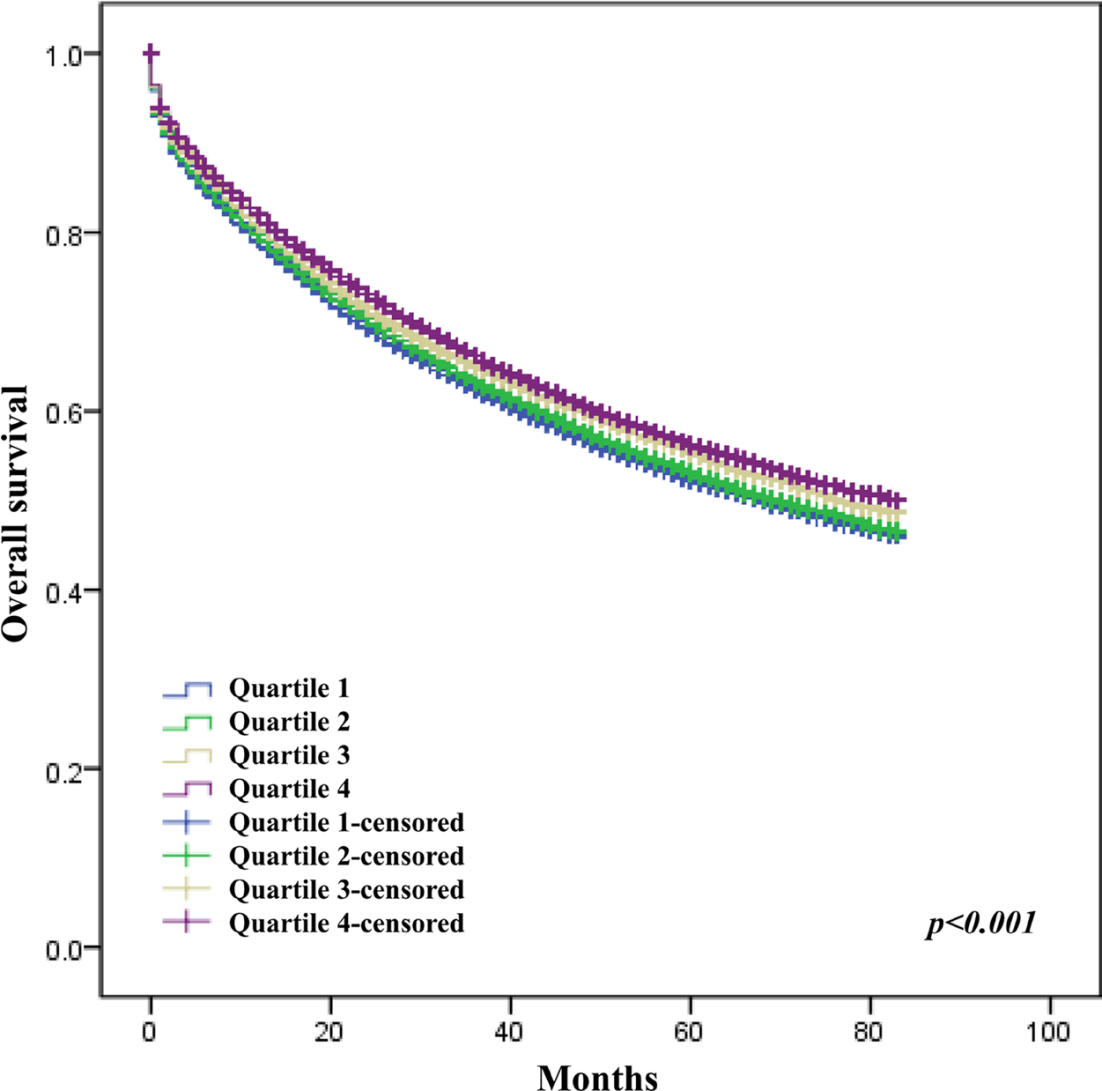
Effect of education level on survival of CRC patients

**Figure 2:**
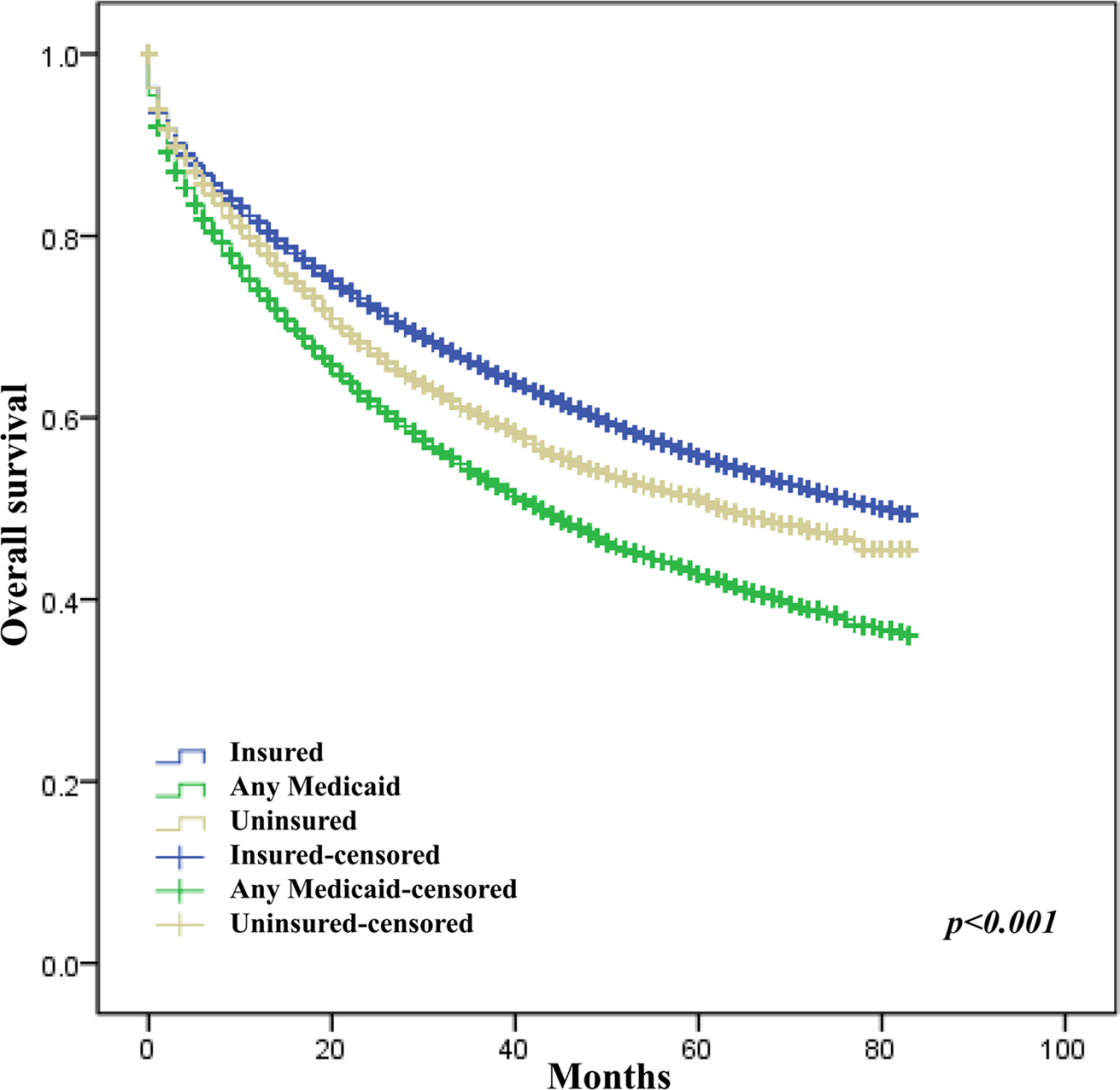
Effect of insurance on survival of CRC patients

**Figure 3:**
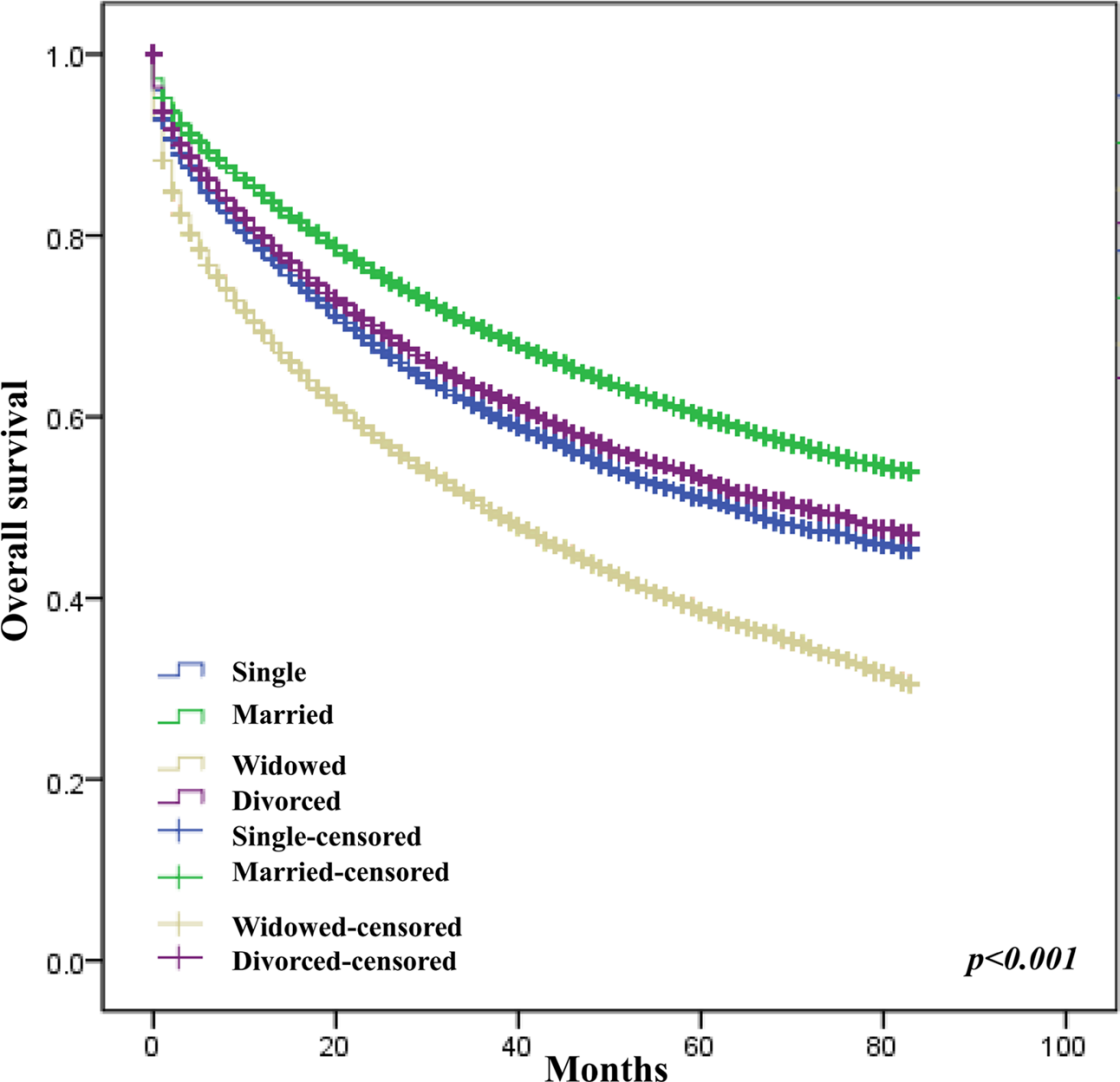
Effect of marital status on survival of CRC patients

**Figure 4:**
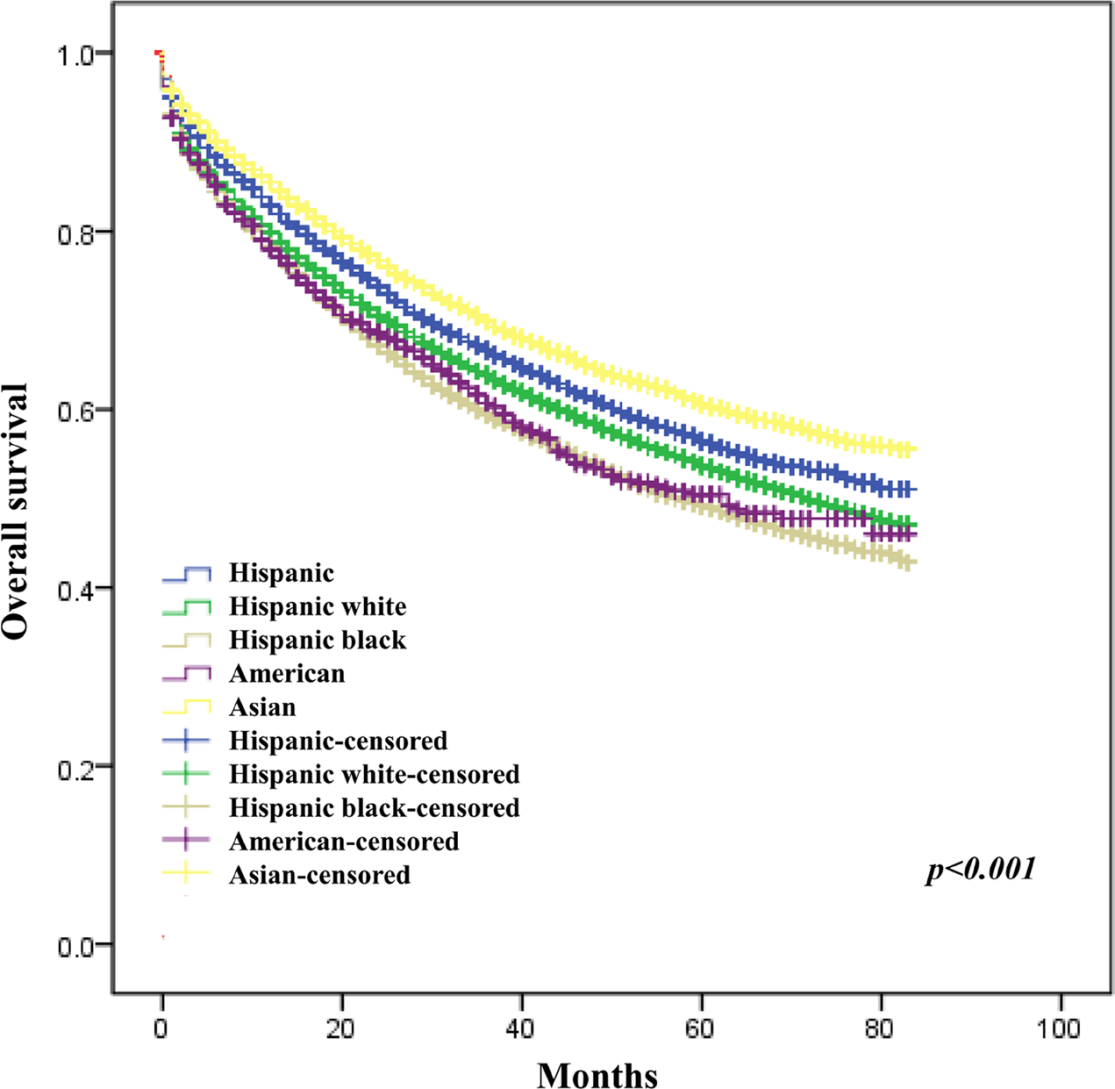
Effect of race on survival of CRC patients

The crude analysis showed that age, tumor site (colon vs rectum, HR = 0.90, 95% CI 0.88–0.91; right vs left colon, HR = 0.95, 95% CI 0.92–0.98), differentiation level, AJCC staging, race, SEER registry, marital status, insurance status, income level and education level were all significantly associated with CRC overall survival (Table 2). After adjustment for confound factors, female sex, age, tumor site (rectum vs colon), differentiation level, AJCC stage, race, SEER registry, marital status, insurance status, and education level were still associated with OS of CRC (Figures 1, 2, 3, 4 and Table 2).

**Table 2:** Factors associated with OS of patients with CRC at the time of diagnosis in the US SEER, 2007 to 2012

Factors	Crude HR (95% CI)	Multivariable HR (95% CI)
**Female sex**	0.96 (0.94–0.97)	0.83 (0.81–0.84)
**Age**		
< 45	1.00 (reference)	1.00 (reference)
45–59	0.99 (0.95–1.04)	1.16 (1.11–1.21)
60–74	1.33 (1.28–1.38)	1.72 (1.62–1.80)
≥ 75	2.63 (2.53–2.74)	3.71 (3.56–3.87)
**Site**		
Rectum VS Colon	0.90 (0.88–0.91)	0.97 (0.95–0.99)
Left VS Right colon	0.95 (0.92–0.98)	0.98 (0.95–1.01)
**Differentiation**		
Well	1.00 (reference)	1.00 (reference)
Moderate	1.33 (1.29–1.37)	1.05 (0.99–1.12)
Poor	2.34 (2.26–2.43)	1.42 (1.33–1.52)
Undifferentiation	2.64 (2.50–2.80)	1.63 (1.48–1.80)
**AJCC stage**		
I	1.00 (reference)	1.00 (reference)
II	1.37 (1.33–1.40)	1.17 (1.12–1.23)
III	1.72 (1.67–1.76)	1.85 (1.76–1.94)
IV	7.29 (7.11–7.47)	7.82 (7.46–8.21)
**Race**		
Hispanic	1.00 (reference)	1.00 (reference)
Non-Hispanic White	1.13 (1.10–1.16)	1.06 (1.03–1.09)
Non-Hispanic Black	1.27 (1.23–1.32)	1.20 (1.15–1.24)
Non-Hispanic American/Alaska native	1.23 (1.12–1.36)	1.12 (1.00–1.26)
Non-Hispanic Asian or Pacific Islander	0.87 (0.84–0.91)	0.89 (0.86–0.93)
Other	0.35(0.28–0.44)	0.53 (0.42–0.66)
**Registry**		
San Francisco-Oakland SMSA	1.00 (reference)	1.00 (reference)
Connecticut	1.07 (1.02–1.13)	0.91 (0.86–0.96)
Detroit (Metropolitan)	1.25(1.20–1.32)	1.03 (0.97–1.09)
Hawaii	0.93 (0.87–0.997)	1.01 (0.94–1.09)
Lowa	1.13 (1.07–1.18)	0.94 (0.89–0.99)
New Mexico	1.12(1.05–1.19)	0.99 (0.92–1.06)
Atlanta (Metropolitan)	1.06 (1.00–1.12)	1.04 (0.97–1.10)
San Jose-Monterey	0.93 (0.87–0.99)	0.92 (0.86–0.98)
Los Angeles	1.07 (1.03–1.12)	0.88 (0.83–0.93)
California excluding SF/SJM/LA	1.07 (1.03–1.11)	0.97 (0.93–1.01)
Kentucky	1.20 (1.15–1.25)	1.04 (0.99–1.10)
Louisiana	1.15 (1.10–1.20)	0.97 (0.92–1.03)
New Jersey	1.21 (1.16–1.26)	0.99 (0.95–1.03)
Greater Georgia	1.12 (1.08–1.18)	1.01 (0.96–1.06)
Utah	0.996 (0.93–1.07)	1.05 (0.98–1.13)
Seattle (Puget Sound)	1.01 (0.96–1.06)	0.94 (0.89–0.99)
Rural Georgia	1.21 (1.02–1.44)	1.02 (0.86–1.21)
**Marital Status**		
single(never married)	1.00 (reference)	1.00 (reference)
Married(including common law)	0.74 (0.72–0.75)	0.74 (0.73–0.76)
Widowed	1.44 (1.40–1.47)	1.04 (1.01–1.07)
Divorced	0.93 (0.90–0.96)	0.92 (0.89–0.95)
**Insurance status**		
Insured	1.00 (reference)	1.00 (reference)
Any Medicaid	1.45 (1.42–1.48)	1.36 (1.33–1.40)
Uninsured	1.17 (1.12–1.22)	1.32 (1.26–1.38)
**County-level income at time of diagnosis**		
Quartile 1 (< US $48,540)	1.00 (reference)	1.00 (reference)
Quartile 2 (US $48,550–55,870)	0.92 (0.91–0.94)	1.03 (0.998–1.06)
Quartile 3 (US $55,870–68,520)	0.91 (0.89–0.93)	0.995 (0.97–1.03)
Quartile 4 (> US $68,520)	0.86 (0.84–0.87)	1.00 (0.96–1.05)
County-level education		
Quartile 1 (< 21.06%)	1.00 (reference)	1.00 (reference)
Quartile 2 (21.07–29.91%)	0.97 (0.95–0.99)	0.96 (0.93–0.98)
Quartile 3 (29.92–35.57%)	0.92 (0.90–0.94)	0.92 (0.89–0.94)
Quartile 4 (> 35.57%)	0.88 (0.86–0.90)	0.87 (0.84–0.90)

Abbreviations: CRC indicates colorectal cancer; SEER, Surveillance, Epidemiology, and End Results.

^a^Counties ranked by median household income in US $10.

^b^Counties ranked by the percentage of adult individuals with a Bachelor's degree.

**Figure 5:**
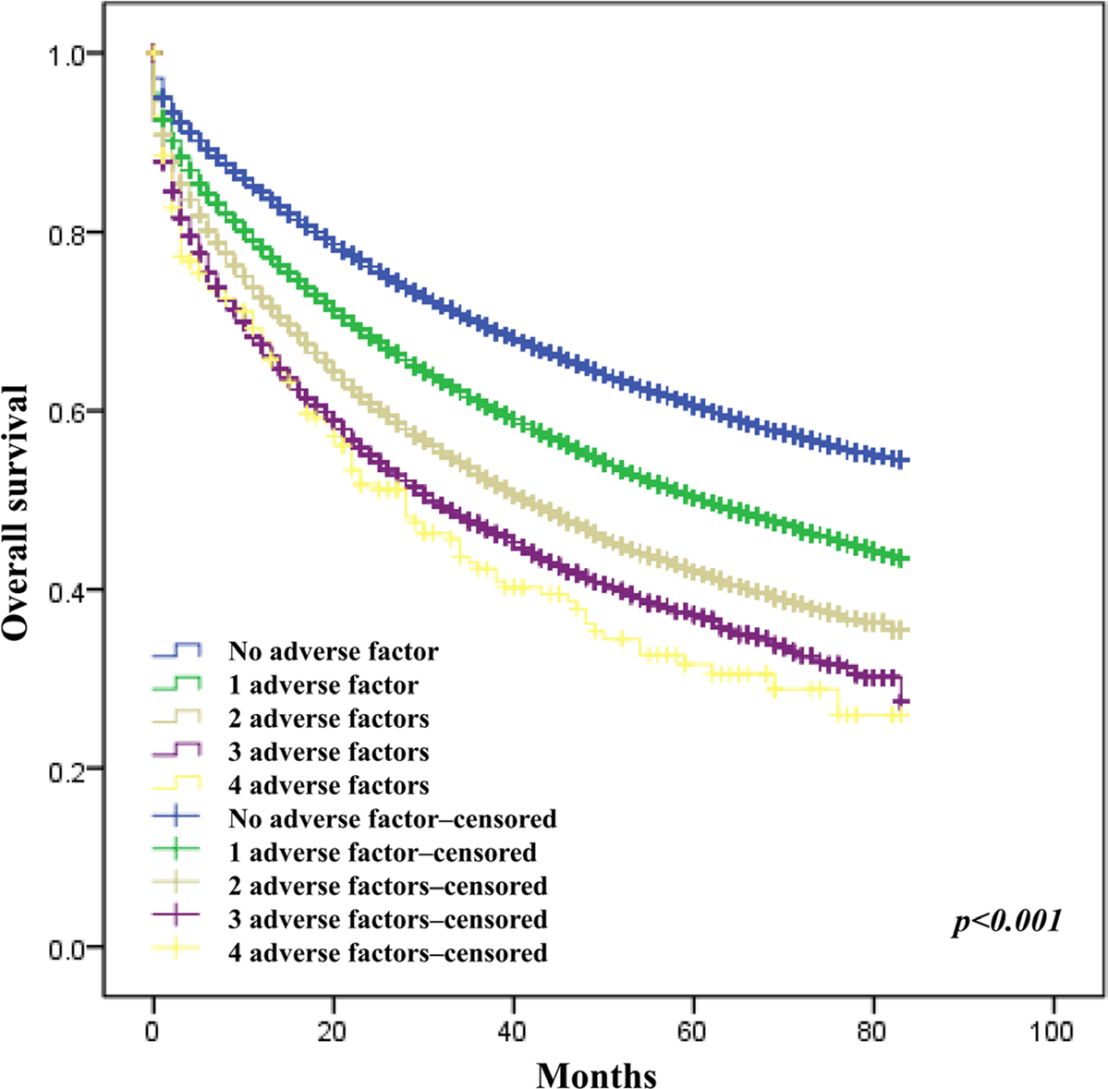
Survival according to the number of adverse SES factors: being widowed, uninsured, Non-Hispanic Black or lowest education level

To validate the impact of SES on CRC survival, we divided these individuals into five groups according to the number of survival-adverse factors. The cumulative effect of SES factors associated with shorter survival in the multivariable analysis (ie, marital status other than married, insurance status other than “insured,” race other than Non-Hispanic Black, and residence in a county within the lowest 1 quartile of education) was shown in Figure 5.

After adjusted for SEER registry, age, and sex, the presence of 1 adverse SES factor (HR, 1.27; 95% CI, 1.25–1.29), 2 adverse SES factors (HR, 1.55; 95% CI, 1.51–1.59), 3 adverse SES factors (HR, 1.80; 95% CI, 1.71–1.90), and 4 adverse SES factors (HR, 2.10; 95% CI, 1.77–2.48) were found to be associated with a gradually higher risk of death when compared with individuals with no adverse SES factors (Table 3).

**Table 3:** Survival analysis according to number of adverse socioeconomic factors-adjusted for age, sex, differentiation, site and stage

	No adverse factors		1 Adverse factor		2 Adverse factors		3 Adverse factors		4 Adverse factors	
	NO. (%)	HR (95%CI)	NO. (%)	HR (95%CI)	NO. (%)	HR (95%CI)	NO. (%)	HR (95%CI)	NO. (%)	HR (95%CI)
**Female Sex**	36035 (40.1)	0.85 (0.81–0.89)	34886 (49.5)	0.87 (0.83–0.90)	12329 (59.5)	0.78 (0.72–0.84)	2081 (68.8)	0.80 (0.67–0.98)	189 (82.9)	0.52 (0.18–1.50)
**Median age (range), y**	65 (20–102)		69 (20–107)		71 (20–108)		72 (21–101)		75 (49–105)	
< 45	6177 (6.9)	1 (reference)	3499 (5.0)	1 (reference)	979 (4.7)	1 (reference)	116 (3.8)	1 (reference)	0	–
45–59	26129 (29.1)	1.21 (1.06–1.38)	16167 (22.9)	1.23 (1.05–1.44)	4415 (21.3)	1.27 (0.99–1.64)	535 (17.7)	0.78 (0.45–1.34)	24 (10.5)	1 (reference)
60–74	33943 (37.8)	1.70 (1.49–1.93)	24633 (35.0)	1.61 (1.39–1.87)	6776 (32.7)	1.87 (1.46–2.38)	1038 (34.3)	1.04 (0.63–1.74)	84 (36.8)	1.24 (0.32–4.79)
≥ 75	23660 (26.3)	3.60 (3.17–4.09)	26152 (37.1)	3.29 (2.84–3.82)	8539 (41.2)	3.44 (2.71–4.38)	1336 (44.2)	1.72 (1.04–2.83)	120 (52.6)	2.26 (0.64–8.95)
**Differentiation**										
Well	7676 (8.5)	1 (reference)	5794 (8.2)	1 (reference)	1686 (8.1)	1 (reference)	255 (8.4)	1.00 (reference)	25 (11.0)	1 (reference)
Moderate	55574 (61.8)	1.04 (0.94–1.15)	43373 (61.6)	1.02 (0.93–1.12)	12764 (61.6)	1.14 (0.99–1.33)	1820 (60.2)	1.12 (0.78–1.61)	151 (66.2)	1.28 (0.37–4.40)
Poor	13270 (14.8)	1.51 (1.36–1.67)	10177 (14.4)	1.38(1.25–1.53)	2777 (13.4)	1.51 (1.28–1.78)	423 (14.0)	1.55 (1.05–2.31)	23 (10.1)	3.06 (0.06–15.58)
Undifferentiation	1616 (1.8)	1.65 (1.42–1.93)	1543 (2.2)	1.62 (1.41–1.87)	445 (2.1)	2.04 (1.62–2.56)	55 (1.8)	1.45 (0.78–2.69)	2 (0.9)	4.03 (0.29–56.47)
Site										
Colon	61730 (68.7)		49495 (70.3)		14801 (71.5)		2157 (71.3)		159 (69.7)	
Right colon	17782 (19.8)		15676 (22.3)		4742 (22.9)		661 (21.9)		51 (22.4)	
Left colon	6922 (7.7)	0.94 (0.89–0.99)	5660 (8.0)	0.95 (0.90–0.999)	1842 (8.9)	1.07 (0.98–1.16)	315 (10.4)	0.95 (0.78–1.15)	23 (10.1)	2.20 (0.96–4.28)
Rectum	24918 (27.7)	0.97 (0.94–0.99)	18067 (25.6)	0.99 (0.96–1.02)	4939 (23.8)	0.97 (0.93–1.02)	696 (23.0)	0.91 (0.80–1.03)	56 (24.6)	1.02 (0.66–1.58)
Large intestine, NOS	2614 (2.9)		2498 (3.5)		873 (4.2)		159 (5.3)		13 (5.7)	
**AJCC stage, no. (%)**										
I	22340 (24.8)	1 (reference)	16321 (23.2)	1 (reference)	4427 (21.4)	1 (reference)	645 (21.3)	1 (reference)	55 (24.1)	1 (reference)
II	21034 (23.4)	1.25 (1.16–1.36)	17329 (24.6)	1.20 (1.11–1.29)	5052 (24.4)	0.98 (0.86–1.11)	756 (25.0)	0.82 (0.60–1.11)	52 (22.8)	0.35 (0.12–0.99)
III	23165 (25.8)	1.98 (1.83–2.15)	17164 (24.4)	1.87 (1.74–2.02)	4813 (23.2)	1.62 (1.43–1.84)	647 (21.4)	1.42 (1.05–1.92)	68 (29.8)	0.43 (0.17–1.11)
IV	16115 (17.9)	9.50 (8.81–10.25)	13432 (19.1)	7.42 (6.89–7.98)	4405 (21.3)	5.92 (5.25–6.68)	657 (21.7)	4.58 (3.41–6.15)	28 (12.3)	0.78 (0.25–2.460)
Unstaged	7255 (8.1)		6205 (8.8)		2012 (9.7)		320 (10.6)		25 (11.0)	

Abbreviations: CRC indicates colorectal cancer.

Furthermore, we evaluated the effect of female sex, age, differentiation level, tumor site (right colon vs left colon, rectum vs colon) and AJCC staging on survival of colorectal cancer in the five groups. The data showed that sex, age, differentiation level, and AJCC staging still exert significant effect on the survival of CRC patients in these groups except in the four-adverse-factor group.

## DISCUSSION

As far as we know, the current research is the largest study to date that evaluates the impact of SES factors on the survival of CRC patients. Our data show that Non-Hispanic Black, being single, divorced or widowed, being uninsured or Medicaid, county-level income are related with an increased risk of death after adjusting for confounding factors including sex, age, SEER registry.

Many studies have detected the relationship between marital status and survival of cancers including CRC [5]. In thyroid cancer [6], gastric cancer [2], pancreatic cancer [3] and colorectal cancer [4], unmarried patients showed poorer prognosis compared with married patients. The reason that marital status is associated with cancer survival is complicated. Unmarried patients might tend to be more depressed and anxious and at higher nonadherence with prescribed treatments than married individuals [7, 8]. The negative emotions impair immune system function and thus may increase the mortality [9]. Aging itself is an adverse prognostic factor in CRC as shown in our result and other studies [10, 11] considering that aging impaired immune system, increased oxidative stress, shortening of telomeres, accumulation of senescent cells [12]. The widowed group contained a very high proportion of elderly patients (data not shown), which might result in the poor survival in this group.

Our study found that the insured patients have the best overall survival compared with uninsured and Medicaid patients. A recent study showed that Medicaid or uninsured status was associated with decreased diagnosis rates of nonpalpable prostate cancer and increased conservative management [13], thus impaired the survival of patients. CRC screening should begin at 50 years of age as it has been shown that screening adults aged 50–75 years reduces CRC mortality. Screening tests for CRC include stool-based tests such as fecal immunochemical test and guaiac fecal occult blood test and direct visualization tests including colonoscopy, CT colonoscopy and flexible sigmoidoscopy [14, 15]. Delayed cancer screen may also affect the survival of cancer patients. Uninsured and Medicaid-insured cancer patients have been shown to present with more advanced disease, less often receive cancer-directed therapy and suffer higher rates of mortality than those with private insurance [16]. For CRC, lack of insurance is associated with an elevated risk of late-stage diagnosis and a decreased likelihood of undergoing screening and receiving treatment following a diagnosis [17]. What's more, in a 2011 survey, nearly one third of US physicians were unwilling to accept new patients with Medicaid insurance. It might cause inappropriate treatment for those Medicaid cancer patients [18]. Until ways are found to provide health insurance to all citizens, it seems likely that the uninsured will continue to suffer poor health outcomes.

We found that the Non-Hispanic Black patients have the worst prognosis compared with the other races. Our findings are consistent with the previous studies. Actually, a number of researches have suggested that the higher mortality rate of Black colorectal cancer patients might be in part due to more invasive tumors and more advanced stage [19–21], treatment strategies variance [22–25], chemotherapy resistance [26], and screening and post-surgery surveillance. Besides, some studies highlighted that racial differences in CRC incidence and mortality could be attributed more to differences in use of health-care than biological disparities [27].

Our study had some limitations. Firstly, the lifestyle information, comorbidities, screening and surveillance tests, or receipt of chemotherapy are not included in SEER database. Secondly, the insurance status recorded at the time of diagnosis could have changed over the follow-up period. Thirdly, some cases are not included in the cohort which may affect the generalizability to the population. Moreover, we were not able to retrieve individual-level SES factors from the SEER Limited-Use data. Census county-level measures are imperfect indicators of individual-level SES [28], and accordingly, our results may be somewhat biased by residual confounding. Lastly, some unknown or unidentified confounders could also impact the analysis.

Despite these potential limitations, our data confirmed that unmarried, Medicaid, lower education level and Non-Hispanic Black CRC patients are at a greater risk of mortality. The physicians, government and health care systems should provide specific cares and interventions for these patients to improve the survival of these patients.

## MATERIALS AND METHODS

### Patient selection in the SEER database

Patients’ data were from the Surveillance, Epidemiology, and End Results (SEER) registry program (http://seer.cancer.gov/data/citation.html). The SEER-18 includes population-based cancer populations reported in the Atlanta, Connecticut, Detroit, Hawaii, Iowa, New Mexico, San Francisco-Oakland, Seattle-Puget Sound, Utah, Los Angeles, San Jose-Monterey, Rural Georgia, Alaska Native, Greater California, Kentucky, Louisiana, New Jersey, and Greater Georgia registries, that represent approximately 28% of the population in the US. The SEER has been widely used in many cancer researches.

Using the SEER-stat software (SEER*Stat 8.3.2), we selected patients that were newly diagnosed with CRC between 2007 (the year when insurance information were available) and 2013 (the most recent year for which data were available) with single primary CRC. The exclusion criteria included that patients at diagnosis were less than 18 years old, had undefined TNM stage, developed more than one primary cancer but the CRC wasn't the first one, had unknown insurance status, marital status, income or education level, had unknown cause of death or unknown survival months. The 7th AJCC staging edition was adopted to redefine stage I, II, III, and IV. Patients with AJCC stage 0 disease were also excluded.

### Socioeconomic factors analysis

The following factors were included and analyzed: age at the time of diagnosis (continuous), gender, race/ethnicity (Hispanic, non-Hispanic white, non-Hispanic black, Non-Hispanic American/Alaska native, Non-Hispanic Asian or Pacific Islander and other), SEER registry, marital status (married, divorced, single, or widowed), insurance status (any Medicaid, insured, or uninsured), median household income (in quartiles), percentage of adult individuals with at least a Bachelor's degree within the county of residence (in quartiles), follow-up time, and vital status (alive or dead).

### Statistical analysis

The χ2 test was adopted to analyze individuals’ basic characteristics appropriately. Cox proportional hazards models were used to calculate the overall survival of CRC patients with the hazard ratio (HR) and corresponding 95% confidence interval (95% CI). The confounding factors including gender, age, education (in quartiles), household income (in quartiles), and race/ethnicity were adjusted. Survival plots were generated using the Kaplan-Meier method. A 2-sided *p* value 0.05 was considered to be statistically significant. All analyses were performed using the statistical software package SPSS for Windows, version 17 (SPSS Inc., Chicago, IL, USA).

## Abbreviations

CRC: Colorectal cancer
SES: socioeconomic, status
HR: hazard ratio
CI: confidence interval
SEER: Surveillance, Epidemiology, and End Results
OS: overall survival
